# LONELINESS AND SOCIAL ISOLATION: TO SCREEN OR NOT? PROVIDERS’ COMMUNICATIONS AND COMFORTABILITY

**DOI:** 10.1093/geroni/igad104.0740

**Published:** 2023-12-21

**Authors:** Su-I Hou

**Affiliations:** School of Global Health Management & Informatics, University of Central Florida, Orlando, FL, USA, Oviedo, Florida, United States

## Abstract

**Purpose:**

Research has shown that loneliness and social isolation (SI) can seriously impact older adults’ health and well-being. However, it is unknown whether healthcare providers communicate with their older patients about these issues. This pilot study examines providers’ communication and comfortability on loneliness screening.

**Methods:**

A convenience sample of providers who see older patients were recruited from healthcare and long-term care settings (n=59). Communication about social isolation was adapted from the revised UCLA 3-item Scale. De Jong Gierveld’s loneliness 6-item scale was used to assess social and emotional loneliness. Comfortable levels with loneliness screening (4 items) were adapted from UCF researchers’ food insecurity screening study.

**Results:**

Providers’ mean age was 46 years; the majority were females (70%), and 67% were whites, with an average of 17 years of practice experience. About 60% of providers believe loneliness screening should be conducted among older patients during every visit. All the scales showed high internal consistencies with Cronbach’s alphas, ranging from .825 to .897. Data showed that communications about loneliness and social isolation were both low, with item means of 2.34 and 2.15, respectively. Overall, providers showed moderate comfort in conducting such screenings (mean=3.72 on a 5-point Likert scale).

**Discussion:**

This pilot provides evidence of high reliabilities of these validated measures to assess providers’ communication and comfortability to screen for loneliness and SI. Data showed concerning findings on low communication and only moderate comfort among providers. It calls attention to providing provider training for early intervention, particularly among older patients.

